# The Practical and Social Functioning (PSF) scale: development and measurement properties of an instrument for assessing activity and social participation among people with serious mental illness

**DOI:** 10.1186/s12888-024-06135-x

**Published:** 2024-10-16

**Authors:** Hanne Clausen, Torleif Ruud

**Affiliations:** 1 Division of Mental Health Services, Akershus University Hospital, Lørenskog, Norway; 2 Norwegian National Advisory Unity On Concurrent Substance Abuse and Mental Health Disorders, Innlandet Hospital Trust, Brumunddal, Norway; 3 Institute of Clinical Medicine, University of Oslo, Oslo, Norway

**Keywords:** Functioning, Severe mental illness, Psychosis, Activity, Participation, Instrument, Measurement, ICF framework

## Abstract

**Background:**

Participation in society and the ability to perform various activities are crucial aspects of everyday functioning. The intertwined relationship between functioning, disability, and health is emphasized in the “International Classification of Functioning, Disability and Health (ICF)” framework. In recent decades, mental health care units have increasingly focused on this aspect. The Practical and Social Functioning scale (PSF) was developed and validated in Norwegian as an easy-to-administer instrument to assess practical and social functioning among patients with serious mental illness in different clinical settings.

**Methods:**

The PSF was developed and revised using data from different Norwegian studies. Data from a total of 562 patients with serious mental illness in different clinical settings were included. The validation process included the evaluation of items into categories by 25 professionals. Development and revision took place in three different stages, and factor analyses were conducted. The quality of the PSF was assessed according to the COSMIN standards for systematic reviews on patient-reported outcome measures.

**Results:**

The final version of the PSF comprises seven subscales, each consisting of four items, resulting in a total of 28 items. These subscales, along with their corresponding items, are loaded onto two factors representing the main dimensions of functioning: activity and participation. Content validity comprises three domains: relevance, comprehensiveness, and comprehensibility. Relevance and comprehensibility were found to be adequate, whereas comprehensiveness was doubtful. Structural validity was adequate, internal consistency was very good, and construct validity was adequate compared to the Global Assessment of Functioning scale. Responsiveness was found to be doubtful in our study with data from an 18-month observation period. Nearly 60% of the items showed a ceiling effect. No items showed a floor effect.

**Conclusion:**

The development and validation of the Norwegian version of the PSF resulted in an instrument consisting of seven subscales and a total of 28 items. The items and subscales assess functioning related to two key factors according to the ICF framework: activity and participation. Our results show that the PSF is an easy-to-administer instrument that may be particularly sensitive for detecting variation among persons with severely impaired functioning.

**Trial registration:**

The study *Implementation of Guidelines for the Treatment of Psychoses* was registered retrospectively on 31 August 2017 at ClinicalTrials.gov (NCT03271242).

**Supplementary Information:**

The online version contains supplementary material available at 10.1186/s12888-024-06135-x.

## Background

People with serious mental illness may experience challenges in performing everyday tasks and assessing patients’ level of functioning, as one of several treatment outcomes, is recommended in international treatment guidelines for psychosis [1, 2]. However, functioning is a broad concept comprising many aspects, including cognitive functioning, daily or practical functioning, social functioning, and other areas. These areas can be further divided into different domains [3].

The World Health Organization (WHO) framework “International Classification of Functioning, Disability and Health (ICF)” emphasizes the intertwined relationships among functioning, disability, and health [4]. The ICF underlines that two of the key components of functioning, are activity (the execution of tasks) and participation (involvement in life situations) [4]. The other three domains are body functioning (physiological functioning, including psychological), body structure (anatomic parts), and environmental factors (the physical, social, and attitudinal environment in which people live). The World Health Organization disability assessment schedule (WHODAS) 2.0 is a measure developed to assess the level of disability according to the ICF framework [5], and both the 36-item and the 12-item-versions have been widely used in health research, including in psychiatry [5]. However, there is no consensus on the gold standard in research concerning people with serious mental illness, and many instruments measuring different concepts or domains of functioning exist. One expert survey [6] and two recent systematic reviews [3, 7] investigated the quality of instruments measuring functional outcomes in persons with mental health problems. As the scopes of these three studies were slightly different, the included instruments only partly overlapped. Some were global questionnaires measuring different outcomes, including a subscale for functioning. Others had a single global score for functioning based on the assessment of different life domains, including the Social and Occupational Functioning Scale (SOFAS) [8] and the Personal and Social Performance Scale (PSP) [9]. Yet others comprised several items assessing various domains of functioning, including the Social Functioning Scale (SFS) [10, 11] and the Life Skills Profile (LSP) [12, 13]. In their review, Long and colleagues found a total of 32 instruments that met their inclusion criteria. Of these, the two most widely used instruments were the SFS and the PSP. The authors concluded that the instruments were qualitatively similar, while the aims, settings, and target population differed and that it was unclear if large, multiple-item scales improved the quality of the measures over single-item scales [3]. They recommended that the aim of the study should guide the choice of instrument. A general finding across the expert survey [6] and the two systematic reviews [3, 7] was the lack of information regarding the psychometric properties of the identified instruments. The PSP received the best overall scores in the quality assessment by Long and colleagues [3] and was the only instrument able to detect significant changes over time. The aggregated data on SPS lacked adequate information on changes over time and satisfactory data on interrater reliability [3]. The SFS is a measure comprising 78 items that assesses functioning in seven domains: Social engagement/withdrawal, Interpersonal communication, Pro-social activities, Recreation, Independence-competence, Independence-performance, and Employment/occupation [10]. The 15-item brief version measures functioning in five of the seven domains, and it has been found to be both reliable and valid [11]. The PSP assesses social functioning in four areas: socially useful activities, personal and social relationships, self-care, and disturbing and aggressive behavior [9]. This scale is similar to the function scale of the split Global Assessment of Functioning scale (GAF, divided into one scale for symptom severity [GAF-S] and one scale for impairment of functioning [GAF-F]) [8, 14], which used to be a mandatory outcome measure in specialized mental health services in Norway for many years. While several instruments measuring symptoms had been used to some extent in mental health services, there was no widely spread instrument measuring functioning except the split GAF scale [15]. However, global scores of functioning based on the assessment of various life domains do not necessarily correlate with patients’ functioning in specific domains. This discrepancy may occur because merging scores from several domains can conceal individual variations in specific areas of functioning [16, 17].

Although many instruments assessing practical and social functioning exist, there was a need for a brief instrument with adequate psychometric properties, to measure the real-life functioning of people with serious mental illness, capturing domains that are important for independent living in the community. The instrument should be easy to administer, require no extensive training, and capture daily life functioning among informants in different settings, including primary care.

### Aims

The aim of this study was to develop and validate a Norwegian instrument that is easily administered for assessing practical and social everyday functioning among persons with serious mental illness and a low to moderate level of functioning. Additionally, this study aimed to evaluate the measurement properties of the instrument and ensure its applicability in both clinical practice and research.

## Methods

### Context

The development, testing, and revisions of the instrument were conducted in community mental health services in Norway. Community Mental Health Centers (CMHCs) provide a broad range of assessments and treatments to the population in their catchment areas. The CMHCs’ outpatient clinics, mobile teams, and inpatient wards serve most of the patients in need of specialized mental health services, including patients with serious mental illness as a core target group. These centers collaborate both with hospital-based mental health services and with community services such as general practitioners and primary mental healthcare [18].

### Design

**Fig. 1:**
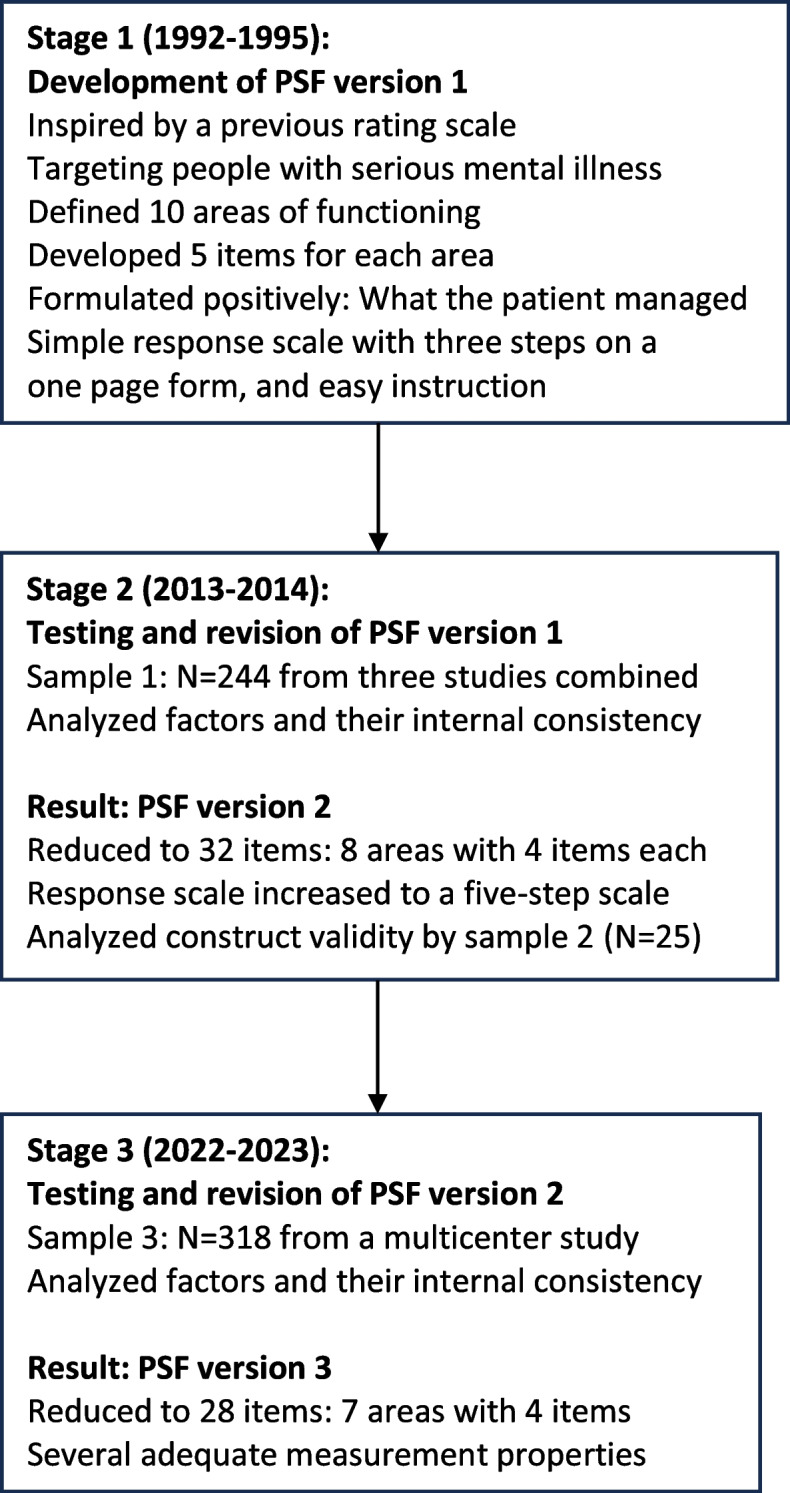
The process of developing, testing, and revising the Practical and Social Functioning scale (PSF)

We conducted the development, testing, and revisions of the Practical and Social Functioning scale (PSF) in three stages as a pragmatic process over many years, as shown in the flowchart (Fig. 1).

The first stage was to develop version 1. In the second stage, we used the instrument in three studies. After conducting psychometric analyses, we were able to reduce the number of items, and concluded with a shortened version 2. In the third stage, we used version 2 in a large multicenter study. The psychometric analyses resulted in version 3. All psychometric analyses and revisions were based on recommendations in the literature on the development and testing of measurement tools [19, 20].

### Stage 1: development of the Practical and Social Functioning scale version 1 (PSF1)

The development of PSF version 1 from 1992–1995 was inspired by the Independent Living Skills Survey developed in the United States [21, 22]. The PSF was designed as an instrument for assessing patients’ level of functioning in ten domains. Items with brief texts described observable behavior and did not require specialist knowledge or expert skills to complete. The assessment required knowledge about the patient and was intended to be completed by a health worker based on observations and information from the patient. It was designed to measure functioning among patients with moderate to low functioning within one or more of the following domains: Care for health, Self-care/clothes, Meals and food, Care for belongings, Managing finances, Use of transportation, Social contact, Conversations, Ability to work, and Leisure activities [23]. For each domain there were five items that covered various aspects and levels of functioning. Each item was positively worded, assessing what the person was able to do, not the level of impairment. Each item had a three-point response scale (0 = not true, 1 = partly true or true part of the time, 2 = true or true all the time) to be easy to assess, and the range of the score for each domain was from 0–10. The scale had a single-page layout, making it easy to use as a clinical instrument. The first five domains (left column on the form) were considered domains measuring practical functioning and giving a total subscore for practical functioning (0–50), and the last five domains (right column) were considered domains measuring social functioning and giving a subscore for social functioning (0–50). The total PSF score ranged from 0 to 100.

The PSF version 1 was included in a system for *Psychiatric Assessment and Follow-up in Municipalities* developed in 1992–1994 in collaboration between the Nordfjord CMHC and the primary mental health services in Eid municipality in the catchment area of the CMHC. The system included a database system built on Epi Info [24] and was acquired by many municipalities throughout Norway to assess, document, and follow up local population needs for mental health and social services as required by a new national law [25]. This wide use of PSF confirmed that it was feasible for use in primary mental health services in the municipalities.

### Stage 2: testing and revision of the Practical and Social Functioning scale version 1 (PSF1)

We tested and revised PSF version 1 from 2013–2014 based on analyses of the measurement properties of the data from sample 1. Sample 1 consisted of completed baseline assessment of all 50 items for 244 patients. Of these, 40 patients (16%) were from a follow-up study by the CMHC where the PSF was developed [23], while 178 patients (73%) were from a national research-based evaluation of the first 12 Assertive Community Treatment (ACT) teams in Norway [26], and 26 patients (11%) were from a smaller study on committed collaborative treatment for patients with serious mental illness. These three studies were conducted at different timepoints. The Nordfjord study took place from 1992–1993 with a seven-year follow-up from 1999–2000. The ACT study took place from 2009–2014, whereas the study on committed collaborative treatment took place from 2011–2015. Sample 1 consisted of 157 males (64%) and 87 females (36%). The mean age was 39 years (standard deviation, SD, 11 years). A total of 55 patients (23%) were younger than 30 years, 86 patients (35%) were aged 30–39 years, 57 patients (23%) were aged 40–49 years, and 46 patients (19%) were aged 50 years or older. The main diagnosis was schizophrenia (*n* = 170 patients, 70%). Other diagnoses included schizoaffective disorder (*n* = 7 patients, 3%), bipolar disorder (*n* = 28 patients, 11%), and severe depression (*n* = 7 patients, 3%), whereas 32 patients (13%) had other severe psychiatric disorders. In all three studies, the PSF was completed by health care workers either together with the patients or based on observations of and information from the patients.

Table 1 shows the decision regarding which items from PSF version 1 were included in PSF version 2.

**Table 1:** Revisions of the Practical and Social Functioning scale (PSF) based on factor analyses^a^

Factor^b^	Item in PSF version 1	Factor loading	Item in PSF2^1^	Item in PSF3^2^	Item in questionnaire
1	B3 Wearing clean clothes and looking clean	0.83	B1	B1	1
	B1 Having good personal hygiene	0.81	B2	B2	2
	B5 Having well-groomed hair (and beard)	0.73	B3	B3	3
	B2 Showering/bathing without help/prompting	0.69	B4	B4	4
	D3 Keeping flat/room clean without help	0.52	-	-	-
	B4 Choosing clothes and dresses without help	0.46	-	-	-
2	E4 Keeping money in a safe place	0.79	D2	D2	10
	E1 Managing own finances	0.78	D1	D1	9
	E5 Making money last until next payment	0.75	D4	D4	12
	E3 Paying own bills/rent/food	0.73	D3	D3	11
	E2 Receiving monthly payment oneself	0.71	-	-	-
	D4 Not destroying own belongings	0.50	-	-	-
	D5 Taking care of own belongings	0.48	-	-	-
3	C1 Making dinner	0.76	C3	C3	7
	C5 Buying/obtaining food	0.67	C1	C1	5
	D1 Washing clothes or having them washed	0.67	C4	C4	8
	C4 Able to follow a recipe	0.66	C2	C2	6
	C3 Making simple meals (sandwiches)	0.56	-	-	-
	D2 Buying clothes without help	0.45	-	-	-
4	J2 Going to movies/concerts/sports/events	0.67	G1	G1	21
	I4 Keeping with a task for 3–4 h	0.55	G4	G4	24
	I1 Working fairly concentrated	0.55	G3	G3	23
	J5 Participating actively in club/society/church	0.52	-	-	-
	J4 Reading books/magazines/newspapers	0.51	-	-	-
	I5 Is interested in having something to do	0.51	-	-	-
	J1 Having hobbies or interests	0.46	G2	G2	22
	C2 Having a fairly varied diet	0.46	-	-	-
	A1 Having usual and fairly health way of living	0.43	-	-	-
	J3 Keeping up with the news in radio/TV	0.33	-	-	-
5	H5 Both staying with a topic and changing the topic	0.77	F2	F2	18
	H3 Listening to others and responding to what they say	0.74	F3	F3	19
	H4 Having ordinary conversations	0.71	F4	F4	20
	H1 Talking distinctly and clearly	0.68	F1	F1	17
	I3 Understanding work instructions fairly well	0.50	-	-	-
	I2 Meets at time and place as agreed upon	0.46	-	-	-
6	G2 Having one or more close friends	0.79	E2	E2	14
	G4 Having friends outside health/social services	0.77	E1	E1	13
	G3 Visiting other people at least monthly	0.72	E3	E3	15
	G5 Being visited by other people at least monthly	0.67	E4	E4	16
	H2 Taking initiatives to conversations with others	0.58	-	-	-
	G1 Talking with others when meeting them in the street	0.58	-	-	-
7	A3 Buying and keeping medication	0.77	A4	-	-
	A5 Taking medication or no need for medication	0.73	A3	-	-
	A2 Making appointments with doctor/dentist	0.63	A2	-	-
	A4 Ability to care for oneself when ill	0.56	A1	-	-
8	F2 Getting around when travelling on his/her own	0.76	H3	H3	27
	F5 Arranging for transportation when needed	0.63	H2	H2	26
	F4 Using public transportation	0.56	H1	H1	25
	F3 Going on vacation to other places	0.53	H4	H4	28
	F1 Having own means of transportation	0.35	-	-	-

^a^Principal component analysis with Kaiser normalization and varimax rotation, ^1^Sample 1; *N* = 244 and ^2^Sample 3: *N* = 318

^b^Subscales: 1 = Personal hygiene, 2 = Money management, 3 = Household chores, 4 = Work and leisure activites, 5 = Communication skills, 6 = Social contact, 7 (removed in PSF3) = Health maintenance, 8 = Transport and travel

Data analyses of measurement properties in this stage included factor analyses of the data from sample 1 and analyses of the internal consistency of the factors. This combined dataset was the first dataset for the PSF large enough to conduct factor analyses to examine whether the scale consisted of the dimensions we aimed for when developing the scale. We also aimed to revise the scale and make it shorter. This included removing items based on the results of the data analyses.

We conducted an explorative principal component analysis with eigenvalues of 1 and varimax rotation, which yielded 11 factors where several factors had only two or three items, and three items loaded on two factors. We subsequently conducted factor analyses for ten, nine, and eight factors. We found that eight factors provided the best solution with only two items loading above 0.40 on more than one factor and with consistency in the content of the items within each factor. We decided to revise the scale, keeping eight factors and reducing the number of items in each scale to four to further reduce the length of the scale. This resulted in a reduction from 50 to 32 items. For each factor, we retained the four items with the highest loading on the factor (except for item J1 of factor 4, which we assessed as more important than three with somewhat higher loading). The eight subscales in PSF version 2 were *Health maintenance, Personal hygiene, Housekeeping chores, Money management, Social contact, Communication skills, Work and leisure activities, and Transport and travel*. Finally, we also revised the three-point response scale to a five-point response scale to increase the variance and sensitivity for the PSF and each subscale.

In stage 2 we also tested the construct validity of PSF version 2 by asking the participants in sample 2 without any previous knowledge of the instrument to sort the questions on the identified factors using a paper form. Sample 2 consisted of 25 researchers, PhD candidates, clinicians, and administrative workers from a department of research and development in mental health service at a university hospital near Oslo, Norway. The participants in this sample had diverse backgrounds relevant to mental health services, including psychiatrists, psychologists, mental health nurses, social workers, and administrative employees. This sample did not complete the PSF 2 for any patients but conducted a validation of the PSF 2 subscales by sorting items on subscales according to content. The construct validity test was conducted in 2013.

The participants sorted each item according to the factor they considered it belonged to [19, 20]. Our hypothesis was that their sorting of the questions would be similar to the sorting made by the factor analysis, examining responses by comparing the distribution (percentage) of each question based on the factors.

Table 2 shows the results from the construct validity test of PSF version 2. For all the items except one, 76–100% of the 25 participants in sample 2 sorted the item into the correct dimension according to the factor structure. The item ‘Going on vacation to other places’ was sorted to the correct dimension by 48%, which was the dimension selected by the largest group of participants. Based on these results, we considered the construct validity of the instrument to be adequate.

**Table 2:** Face validity sorting items (%) on subscales of the PSF2 (25 raters)

	**A**	**B**	**C**	**D**	**E**	**F**	**G**	**H**	**Missing**
**A. Health maintenance**									
A1. Look after oneself when ill	**100**	0	0	0	0	0	0	0	0
A2. Making appointments with doctor/dentist	**84**	0	0	0	0	8	4	0	4
A3. Taking medication or no need for medication	**96**	0	0	0	0	0	0	0	4
A4. Buying and keeping medication	**100**	0	0	0	0	0	0	0	0
**B. Personal hygiene**									
B1. Wearing clean clothes and looking clean	4	**92**	4	0	0	0	0	0	0
B2. Having good personal hygiene	4	**96**	0	0	0	0	0	0	0
B3. Having well-groomed hair (and beard)	8	**88**	4	0	0	0	0	0	0
B4. Showering/bathing without help/prompting	4	**96**	0	0	0	0	0	0	0
**C. Household chores**									
C1. Buying/obtaining food	0	0	**80**	16	0	0	4	0	0
C2. Able to follow a recipe	0	0	**80**	0	0	0	20	0	0
C3. Making dinner	8	4	**80**	0	4	0	0	0	4
C4. Washing clothes or having them washed	8	16	**76**	0	0	0	0	0	0
**D. Money management**									
D1. Managing own finances	0	0	0	**96**	0	0	0	0	4
D2. Keeping money in a safe place	4	4	0	**92**	0	0	0	0	0
D3. Paying own bills/rent/food	0	0	4	**96**	0	0	0	0	0
D4. Making money last until next payment	0	0	0	**96**	0	0	0	0	4
**E. Social contact**									
E1. Having friends outside health/social services	0	0	0	0	**92**	4	4	0	0
E2. Having one or more close friends	0	0	0	4	**84**	8	0	0	4
E3. Visiting other people at least monthly	0	0	0	0	**88**	8	0	0	4
E4. Being visited by other people at least monthly	0	0	0	0	**84**	13	0	0	4
**F. Communication skills**									
F1. Talking distinctly and clearly	0	0	0	0	0	**100**	0	0	0
F2. Both staying with a topic and changing the topic	0	0	0	0	0	**100**	0	0	0
F3. Listening to others and responding to what they said	0	0	0	0	4	**96**	0	0	0
F4. Having ordinary conversations	0	0	0	0	0	**100**	0	0	0
**G. Work and leisure activities**									
G1. Going to movies/concerts/sports/events	0	0	0	0	12	0	**80**	4	4
G2. Having hobbies or interests	4	0	0	0	4	0	**92**	0	0
G3. Working fairly concentrated	12	0	0	0	0	0	**88**	0	0
G4. Keeping with a task for 3–4 h	8	0	0	0	0	4	**80**	0	8
**H. Transport and travel**									
H1. Using public transportation	0	0	0	0	0	0	0	**100**	0
H2. Arranging for transportation when needed	0	0	0	0	0	8	0	**88**	4
H3. Getting around when travelling on his/her own	0	0	0	0	0	0	8	**92**	0
H4. Going on vacation to other places	0	0	0	0	4	0	44	**48**	4

The data analyses in stage 2 were conducted with IBM SPSS (Armonk, NY, USA) for Windows version 23.

### Stage 3: testing and revision of the Practical and Social Functioning scale version 2 (PSF2)

We tested and revised PSF version 2 from 2022–2023 based on analyses of the measurement properties (factor structure and internal consistency of factors) of the baseline data from sample 3. Sample 3 consisted of data from 318 patients included in a multicenter study of patients with psychosis in treatment in 39 clinical units (CMHCs, hospital departments) in six health trusts throughout urban and rural areas in Norway. The study took place from 2016–2017. Sample 3 consisted of 188 males (59%) and 130 females (41%). The mean age of the participants was 40 years (SD 13 years). Among them, 78 (25%) under the age of 30 years, 87 (27%) were aged 30–39 years, 76 (24%) were aged 40–49 years, and 77 (24%) were aged 50 years or above. The main diagnosis was schizophrenia (*n* = 156 patients, 49%). Other diagnoses included schizoaffective disorder (*n* = 59, 19%), bipolar disorder (*n* = 15, 5%), severe depression (*n* = 4, 1%), and other severe psychiatric disorders (*n* = 84, 26%). The PSF was completed by health care workers either together with the patients or based on observations of and information from the patients.

The subscales *Money management, Social contact, Communication skills, Work and leisure activities,* and *Transport and travel* were found to be separate factors and were kept with four items each. These factors were shown to be robust factors across samples. The items in *Health maintenance* (factor A) were distributed with one item in *Personal hygiene* (factor B) and three items in *Household chores* (factor C).

Owing to the changed factor structure in this sample, we decided to make a version 3 (Table 1). Aiming to keep four items in each factor as explained above, we assessed which items to keep for the new factors B and C by inspecting factor loadings, impact on Cronbach's alpha if an item is removed, and thematic assessment of which items fit best together. Our assessment showed that the original items in factors B and C were the most robust and stable items in these factors, leading to the removal of the items that had been added to factors B and C from factor A. In addition to removing one subscale (four items) as described, we changed the sequence of the subscales in the instrument, as well as the sequence of items in each subscale, according to what we considered a natural sequence of subscales and of items within each subscale. The final numbering is shown in Table 1. We also removed the letters in the numbering of items and renumbered the items in sequence for the entire instrument across subscales. We concluded that the results from the construct validity test in stage 2 remained valid for the new version, as we only removed all the items from one factor and did not make any changes to the other factors or items.

### Data analyses of the final Practical and Social Functioning scale version 3 (PSF3)

#### Content validity

Given that we were not aware of any standards for reporting the measurement properties of instruments measuring functioning, we used the COnsensus-based Standards for the selection of health Measurement INstruments (COSMIN), the COSMIN standards for systematic reviews of patient-reported outcome measures (PROMs), to assess and report content validity, structural validity, internal consistency, and construct validity [27–30]. We assessed content validity using the ten COSMIN criteria for content validity [30]. These criteria are grouped into three main concepts: relevance (five criteria), comprehensiveness (one criterion), and comprehensibility (four criteria). Each item is rated on a 4-point scale (very good, adequate, doubtful, and inadequate). Both authors rated all ten items individually before reaching a consensus.

#### Structural validity and internal consistency

For sample 3 in stage 3 we imputed 79 missing responses (0.78%) with the mean value of each item and conducted an exploratory factor analysis of the items to examine the factor structure of the final scale [19, 20, 31]. We used principal component analysis with varimax rotation and Kaiser’s criterion with an eigenvalue of more than 1, including questions with loadings of 0.40 or more in the factor with the highest loading of the question. We then calculated Cronbach's alpha for each factor and interpreted the degree of internal consistency based on the suggested guidelines as unacceptable (alpha below 0.70), fair (alpha 0.70 to 0.79), good (alpha 0.80 to 0.89), or excellent (alpha 0.90 and above) [32]. We analyzed the corrected item-total correlation for all the items, considering a Pearson correlation of 0.30 or above as adequate [33].

#### Two dimensions

To examine whether the final instrument, PSF version 3, contained the two main dimensions of practical and social functioning as intended when developing PSF version 1, we also conducted a factor analysis requesting two factors. We proceeded in this way for the 28 items as well as for the 7 subscales, considering factor loadings of 0.40 or above as required for both the analysis of items and the analysis of subscales. To examine the strength of the relationship between items and subscales were adequate for factor analyses, we used the Kaiser–Meyer–Olkin Measure of Sampling Adequacy (adequate if > 0.70) and the Barlett’s Test of Sphericity (adequate if < 0.05) [31].

### Construct validity

We assessed the construct validity of PSF version 3 subscales and the two dimensions by analyzing the Pearson correlations of these scales with the GAF-F subscale for 280 patients rated by the clinicians assessing sample 3 at baseline. Both PSF version 3 and the GAF-F measure the level of functioning. However, the PSF subscales and dimensions consist of groups of items that focus on functioning in specific domains, whereas the GAF-F provides a global score based on the assessment of functioning across several life domains. As these measures have related but dissimilar constructs, correlations should be between 0.30 and 0.50 to consider the construct validity adequate [29].

### Responsiveness

To examine the responsiveness of the PSF version 3, we used a paired samples test to analyze changes in PSF scores from baseline to the 18-month follow-up for 104 patients in sample 3. We analyzed changes in scores for the seven subscales, the two main dimensions, and the total mean score.

### Floor and ceiling effects

We used descriptive statistics to inspect the distribution of responses from sample 3 on all items and subscales in the final instrument. We analyzed floor and ceiling effects of all items with 50% as the cutoff criterion, considering a question acceptable if fewer than 50% of the patients had chosen the most negative or the most positive answer, respectively [34, 35].

The data analyses in stage 3 were performed with IBM SPSS (Armonk, NY, USA) for Windows version 28.

## Results

### Content of the Practical and Social Functioning scale version 3 (PSF3)

The final version of the instrument measures two dimensions of functioning, practical (activity) and social (participation). These two dimensions comprise various life domains. The items that demonstrate commonality in different domains, as shown in the factor analyses, are organized into seven subscales: *Personal hygiene, Household chores, Money management, Social contact, Communication skills, Work and leisure activities,* and *Transport and travel*. Each subscale comprises four items (Table 3). Each item is rated on a five-point response scale.

**Table 3:** Factor analysis^a^, internal consistency of factors (subscales), and corrected item-total correlation for items of Practical and Social Functioning scale version 3 (*N* = 318)

	**1**	**2**	**3**	**4**	**5**	**6**	**7**	**Corrected item-total correlation**
**B. Personal hygiene**								
B2. Having good personal hygiene	**0.88**	0.14	0.12	0.09	0.16	0.10	0.09	0.873
B1. Wearing clean clothes and looking clean	**0.86**	0.15	0.08	0.07	0.16	0.02	0.11	0.816
B3. Having well-groomed hair (and beard)	**0.83**	0.20	0.12	0.12	0.11	0.12	0.03	0.803
B4. Showering/bathing without help/prompting	**0.80**	0.12	0.18	0.05	0.13	0.18	0.08	0.777
**F. Communication skills**								
F2. Both staying with a topic and changing the topic	0.16	**0.83**	0.16	0.05	0.15	0.06	0.17	0.804
F3. Listening to others and responding to what they say	0.11	**0.81**	0.24	0.07	0.03	0.13	0.17	0.771
F4. Having ordinary conversations	0.13	**0.81**	0.13	0.14	0.12	0.11	0.14	0.766
F1. Talking distinctly and clearly	0.22	**0.72**	0.06	0.12	0.15	0.08	0.06	0.627
**D. Money management**								
D1. Managing own finances	0.12	0.06	**0.87**	0.03	0.13	0.09	0.10	0.776
D3. Paying own rent/bills/food	0.04	0.08	**0.72**	0.08	0.33	0.19	0.10	0.631
D2. Keeping money in a safe place	0.28	0.22	**0.71**	0.02	0.10	0.10	0.14	0.654
D4. Making money last until next payment	0.10	0.25	**0.67**	0.07	0.10	-0.01	0.07	0.523
**E. Social contact**								
E1. Have friends outside health services	0.01	0.15	0.10	**0.82**	0.17	0.15	0.06	0.722
E2. Having one or more close friends	0.00	0.14	0.16	**0.80**	0.21	0.16	0.01	0.692
E4. Being visited by other people at least monthly	0.15	-0.01	-0.02	**0.71**	-0.08	-0.11	0.16	0.473
E3. Visiting other people at least monthly	0.11	0.15	-0.05	**0.70**	0.14	0.19	0.12	0.598
**C. Household chores**								
C3. Making dinner	0.11	0.11	0.17	0.11	**0.84**	0.11	0.16	0.743
C1. Buying/obtaining food	0.16	0.20	0.21	0.17	**0.75**	0.15	-0.03	0.631
C2. Able to follow a recipe	0.23	0.10	0.24	0.08	**0.60**	0.09	0.26	0.577
C4. Washing clothes or having them washed	*0.44*	0.11	0.08	0.12	**0.56**	0.09	0.12	0.533
**H. Transport and travel**								
H1. Using public transport	0.08	-0.05	-0.10	0.08	0.11	**0.81**	0.12	0.503
H3. Getting around when travelling on your own	0.12	0.26	0.16	0.06	0.03	**0.71**	0.10	0.532
H2. Arranging for transportation when needed	0.20	0.26	0.25	0.14	0.13	**0.69**	0.12	0.631
H4. Going on vacation to other places	0.06	0.02	0.17	0.20	0.24	**0.47**	0.24	0.409
**G. Work and leisure activities**								
G3. Working fairly concentrated	0.07	0.31	0.12	0.08	0.12	0.16	**0.78**	0.675
G4. Keeping with a task for 3–4 h	0.08	0.21	0.03	0.04	0.16	0.14	**0.77**	0.566
G2. Having hobbies or interests	0.08	0.07	0.17	0.19	0.06	0.10	**0.68**	0.520
G1. Going to movies/concerts/sports/events	0.23	-0.10	0.11	0.40	0.09	0.25	**0.43**	0.390
**Proportion of total variance explained**	31.3%	8.4%	7.5%	6.5%	5.6%	4.6%	4.3%	
**Internal consistency (Cronbach’s alpha)**	0.92	0.80	0.82	0.81	0.88	0.74	0.71	

^a^Principal component analysis with Kaiser criteria of eigenvalues 1 or more and with varimax rotation for the 28 items of Practical and Social Functioning scale 3 with response scale 1–5

The instrument is easy to understand and feasible to use without any extensive training. The PSF has been translated into English with minor adjustments after back translation to Norwegian. The English version is available as online supplementary material.

### Content validity

Our assessment of content validity was based on the ten COSMIN criteria, which assess three domains: relevance, comprehensiveness, and comprehensibility [30]. One of the authors, Torleif Ruud, had extensive communication with health care workers and patients across the different studies and received feedback on the use and content of the rating scale. We also investigated the response rates for all the items. Both findings were included in the evaluation of content validity. We found the instrument *relevant* (for the construct of interest, for the target population, for the context, with an appropriate response scale, and with an appropriate recall period), and *comprehensible* for the patients (understandable instructions, understandable questions and response scales, appropriately worded questions, and with a response scale matching the questions). However, we were uncertain regarding *comprehensiveness*, as there are other, and perhaps newer, key concepts that are not included (e.g., use of internet and social media). We concluded that the relevance and comprehensibility were both adequate, whereas the comprehensiveness was doubtful, as newer concepts emerged after the initial development of the PSF.

### Structural validity and internal consistency

The final scale had adequate structural validity, with seven factors from the 28 items, as shown in Table 3. Only one item (C4) loaded on two factors. The internal consistency was excellent (Cronbach’s alpha 0.90 or above) for one factor, good (alpha 0.80 to 0.89) for four factors, and fair (alpha 0.70 to 0.79) for two factors [32]. As shown in Table 3 (last column), the corrected item-total correlation was above 0.40 for all the items, which is considered adequate [33]. According to the COSMIN criteria, the internal consistency was found to be very good [28–30].

#### Two main dimensions

Table 4 shows that in the factor analysis requesting two factors, each item loaded (0.40 or more) exclusively on one of two factors (upper part of the table), as did each subscale (lower part of the table). The first factor contained all the items of the following subscales: *Personal hygiene, Household chores, and Money management,* and *Communication skills*. The second factor contained all items of the subscales *Social contact, Work and leisure activities and Transport and Travel.* This suggests that the instrument comprises items that align with one of two different dimensions.

**Table 4:** Factor analysis^a^ of Practical and Social Functioning scale version 3 exploring two main dimensions (*N* = 318)

Items	Factor A	Factor B
B2 Having good personal hygiene	**0.78**	0.03
B1. Wearing clean clothes and looking clean	**0.75**	0.00
B4 Showering/bathing without help/prompting	**0.75**	0.04
B3 Having well-groomed hair (and beard)	**0.75**	0.05
D2. Keeping money in a safe place	**0.65**	0.15
F2. Both staying with a topic and changing the topic	**0.61**	0.25
F3. Listening to others and responding to what they say	**0.56**	0.27
C4. Washing clothes or having them washed	**0.56**	0.24
D1. Managing own finances	**0.55**	0.17
F4. Having ordinary conversations	**0.54**	0.32
F1. Talking distinctly and clearly	**0.54**	0.24
C2. Able to follow a recipe	**0.52**	0.32
C1. Buying/obtaining food	**0.50**	0.35
D4. Making money last until next payment	**0.50**	0.16
D3. Paying own bills/rent/food	**0.49**	0.30
C3. Making dinner	**0.47**	0.39
E1. Having friends outside health/social services	0.07	**0.76**
E2. Having one or more close friends	0.10	**0.75**
E3. Visiting other people at least monthly	0.10	**0.68**
G1. Going to movies/concerts/sports/events	0.21	**0.54**
E4. Being visited by other people at least monthly	-0.04	**0.51**
G3. Working fairly concentrated	0.36	**0.51**
H4. Going on vacation to other places	0.25	**0.49**
G2. Having hobbies or interests	0.24	**0.48**
H2. Arranging for transportation when needed	0.47	**0.47**
G4. Keeping with a task for 3–4 h	0.31	**0.45**
H1. Using public transportation	0.12	**0.43**
H3. Getting around when travelling on his/her own	0.36	**0.40**
**Subscales**	**Factor A**	**Factor B**
D. Money management	**0.762**	0.144
B. Personal hygiene	**0.749**	0.142
C. Household chores	**0.717**	0.354
F. Communication skills	**0.645**	0.296
E. Social contact	0.063	**0.837**
G. Work and leisure activities	0.341	**0.707**
H. Transport and travel	0.372	**0.635**

^a^Principal component analysis with varimax rotation with Kaiser normalization and extracting two factors. Kaiser–Meyer–Olkin Measure of Sampling Adequacy 0.876 for items and 0.860 for subscales. Barlett’s Test of Sphericity < 0.001 for items and subscales

The internal consistency of both factors was good, with Cronbach’s alphas of 0.89 and 0.83, respectively.

### Construct validity

We compared the PSF version 3 scores and the GAF-F scores for 280 patients in sample 3 and found that the Pearson correlation coefficient was adequate (0.39 and 0.39) for two subscales (*Household chores, Work and leisure activities*) but doubtful (0.21 to 0.29) for the other five subscales. The Pearson correlation coefficient was adequate (0.36 and 0.41) for the two dimensions (activity and participation), as was the PSF total mean score (0.44). This indicates a small to moderate degree of correlation between these two instruments. Based on these results construct validity was found to be adequate.

### Responsiveness

The analyses of responsiveness using paired samples tests of changes in the PSF version 3 over 18 months for 104 patients in sample 3 showed no significant change in the total mean score for the PSF, or the mean scores for the main dimensions: activity (execution of tasks) and participation (involvement in life situations). The subscale *Communication skills* was the only subscale that showed a small but significant change (mean 0.15, SD 0.70, *p* = 0.030). Responsiveness was found to be doubtful according to the COSMIN criteria.

### Floor and ceiling effects

As shown in Supplementary table A, the frequency distribution for the 28 items on a five-point response scale, which was based on data from 318 participants, showed that more than 50% of the population reported the highest score on 16 items (57%), whereas no floor effect was detected (50% or more reporting the lowest score). For the subscales *Personal hygiene* and *Communication skills*, all the items had over 50% rating the highest score, while the subscales *Household chores* and *Money management* comprised three of four items where more than 50% rated the highest score. The subscale *Transport and travel* comprised two items where more than 50% rated the highest score. The subscales *Social contact* and *Work and leisure activities* comprised no items where more than 50% rated the highest score.

## Discussion

### The final version of the Practical and Social Functioning scale (PSF)

The final version of the PSF comprises seven subscales with four items each. The subscales measure the level of functioning in seven life domains: *Personal hygiene, Household chores, Money management, Social contact, Communication skills, Work and leisure activities,* and *Transport and travel.* The subscales and the corresponding items loaded on one of two factors. These two factors represent two dimensions. The first factor comprises four subscales with the corresponding items that measure the level of ability to perform various activities (practical dimension). The second factor comprises three subscales with corresponding items that measure functioning in areas that are important for participation in the community (social dimension). These two dimensions correspond well with the key components of functioning in the ICF framework: activity (the execution of tasks) and participation (involvement in life situations) [4]. In contrast to the WHODAS 2.0, which was developed to conceptualize the ICF framework and assess the level of impairments in different areas, the PSF measures the ability to perform activities and the level of participation. Therefore, the PSF aligns well with a strength-based approach instead of a problem-focused approach.

### Methodological considerations

The content of the final version of the instrument is the result of a validation process that included data from 562 patients in different clinical settings and 25 professionals at a research department of a large university hospital. The quality of the PSF was assessed using the COSMIN standards for systematic reviews of patient-reported outcome measures (PROMs) [28–30]. This is in line with the quality assessment criteria used by Long and colleagues [3].

Our results show that two of the three domains of content validity were adequate. The structural validity was adequate, and the internal consistency was very good. The construct validity was adequate, whereas responsiveness was doubtful. Long and colleagues reported that studies on the WHODAS scored 1 of 18 points, indicating poor quality missing data. The quality of the data for the SFS was moderate (8 of 18 points), whereas the quality of data for the PSP was good (11 of 18 points) [3].

According to the COSMIN guideline, the content validity of the instrument is evaluated by assessing multiple parts, including general design requirements and the following three aspects: *relevance, comprehensiveness, and comprehensibility*. The ratings are based on subjective judgment [28–30]. The construct “functioning” and the theoretical framework were clearly described, as were the target population and the context of use. All three studies were performed in samples that represent the PSF’s target population. The *relevance* for the construct of interest for the target population and for the context of use were rated as sufficient. The response options and the recall period were also rated as sufficient. *Comprehensibility* assesses whether the instructions, items, and response options are understandable for the target population; whether the items are properly worded; and whether the response options match the items. These items were also rated as sufficient. All the ratings were based on oral communication with professionals and patients in the different studies, and the response rates for the longer versions of the instrument in all the studies were high. Both the feedback and the response rates suggest that the instrument is appropriate for assessing everyday functioning among persons with severe mental illness and moderate to low levels of functioning, that it is acceptable for use by both patients and clinicians, and that it is feasible for use in different service settings [7]. The *comprehensiveness* aspect assesses whether all relevant key concepts are included in the instrument. This was rated insufficiently as the instrument does not include items regarding the use of social media or the internet or sexual functioning, areas that are important for functioning [3]. In the review by Long and colleagues both the SFS and the PSP scored 1, indicating that there was a lack of information regarding content validity or that there were some questions regarding the methodology in the included studies [3].

The PSF is based on a reflective model, and factor analyses were performed. Our results showed that the structural validity of the instrument is adequate. With a sample size of 318 participants and 28 questions (sample/questions 318:28), the ratio was 11:1, which is well above the recommended ratio in the COSMIN guideline. The seven subscales had Cronbach’s alphas between 0.71 and 0.92. According to the COSMIN criteria, the internal consistency for the PSF is sufficient. This is similar to the scores of the SFS and the PSP in the review by Long and colleagues [3]. A subsequent step was to examine the construct validity. In our study, we used the GAF-F subscale as a global estimate of the participants’ level of functioning and the PSF scale to obtain a more detailed assessment. When the scores of these two instruments were compared, the Pearson correlation coefficients were adequate for the two dimensions and for the PSF total mean score. This suggests adequate construct validity; however, further studies should be undertaken to explore construct validity.

An important ability of a questionnaire is the sensitivity to detect clinically meaningful changes over time. In their systematic review of social functioning outcome measures, Long and colleagues concluded that there is a lack of information regarding responsiveness to change and floor-ceiling effects [3]. We did not detect any significant changes in the PSF total mean scores when comparing baseline to the 18-month follow-up among participants in sample 3 or in the mean scores of the two dimensions, activity and participation. However, we did find a small but significant reduction in the mean score of the subscale *Communication skills*. The change indicates a minor reduction in functioning at the 18-month follow-up in this domain, but due to limited data, it is unclear if this reflects true clinical worsening. In two previous papers that included data from sample 1, we also analyzed changes in PSF scores. The first study investigated changes in PSF subscale scores after seven years among persons with severe mental illness (*n* = 40) in a rural setting. All but one subscale showed significantly higher scores indicating better practical and social functioning [23]. However, the study of patients treated by ACT teams (part of sample 1, *n* = 142) showed no significant changes in the total mean score of the PSF after two years [36], which is in line with data reported on the SFS [3]. These findings may suggest that it takes time to achieve significant clinical changes in practical and social functioning among people with severe mental illness. Another possible explanation is that the instrument is not sensitive to small changes in practical and social functioning, although they may be meaningful to patients. In contrast, the PSP scores have been shown to be sensitive to changes [3]. Perhaps the changes are more likely to be detected when using global scores than when using the sum of scores for different domains or areas. Further research is needed to investigate the PSF’s sensitivity to change.

The frequency distribution showed that more than half of the participants reported the highest score for 16 of the 28 items, indicating a ceiling effect on 57% of the items. However, none of the items in the two subscales, *Social contact* and *Work and leisure activities* showed a ceiling effect. No items showed a floor effect. Further research is warranted before conclusions can be drawn, but these findings may suggest that the instrument is particularly sensitive to significantly impaired real-life functioning among persons with severe mental illness in line with the intended quality of the PSF.

Compared with existing instruments measuring social functioning, the PSF may be an important alternative, depending on the scope of the study. The PSF is a brief instrument that captures important aspects of daily life functioning among people with serious mental disorders. It has adequate psychometric properties, is strength-focused, and easy to use in different clinical settings without extensive training.

### Potential use in clinical practice and research

The PSF is freely available with a Creative Commons license. This license allows for free noncommercial use in its present form when citing this article, but it does not permit any commercial use. Both the PSF scale and the user guideline are available in the online supplementary material.

In clinical work, the PSF can provide useful information regarding everyday functioning in different areas among persons with severe mental illness. It covers basic living skills important for living independently. The instrument does not require extensive training or expertise and can therefore be useful in different service settings and used by multiple informants. Due to the ceiling effect that was shown in our analyses, the instrument may be particularly appropriate for detecting severely impaired functioning in real-life settings and determining which areas to prioritize in individual treatment planning. Furthermore, the PSF is positively worded, focusing on abilities, not impairments in contrast to the WHODAS. The statements in the PSF are also generic, and perhaps the rating scale can be useful in other clinical settings providing services for people with severe practical and social impairments, such as dementia or substance use problems.

Earlier studies have shown a positive association between level of functioning and satisfaction with life [26], and between level of functioning and personal recovery [37]. This suggests that practical functioning is an important area that mental health services and policymakers should emphasize in treatment. In addition to the clinical use of instruments to assess functioning among individuals in treatment, standardized assessment of functional outcomes has an important role in mental health services’ routine outcome monitoring as a part of their quality improvement work, e.g., the flexible assertive community treatment model [38–40].

The instrument’s instructions state that it is necessary to have good knowledge of the individual patient’s level of functioning and that it should be used after observation in real-life settings. Preferably, the instrument should be completed together with the patient. The feedback from professionals and participants in these studies suggests that the PSF could also be used as a self-report questionnaire, which would be useful, as PROMs assessing the level of functioning are lacking [41]. However, further research is needed to investigate the validity of the instrument as a PROM.

The instrument may also be valuable in research projects to assess the everyday living skills necessary to live an independent life for persons with severe mental illness. Previous reviews have emphasized the lack of knowledge regarding the various instruments’ responsiveness and sensitivity to change [42, 43]. Particularly, this relates to the sensitivity of changes that are important to patients but are not observed or appreciated by the clinician [3]. Further studies should be undertaken to explore these aspects. It would also be interesting to investigate patients’ view of the ceiling effect in a qualitative study. Additionally, the validity of the PSF against other factors of real-life functioning needs further investigation. Importantly, this study investigated the validity of the Norwegian version. Future studies should examine the psychometric properties of the English version. Content validity should also be assessed by external experts, and further research should test reliability. The high response rate to the instrument in the different studies may suggest that it was acceptable and feasible to use, but appropriate studies are needed. Finally, to use the instrument as a PROM, a study should be conducted to investigate service users’ opinions regarding domains (comprehensiveness) and comprehensibility.

### Strengths and limitations

One of the strengths of this study is that the instrument was developed through a process that included experience from several studies involving people with serious mental illness in different clinical settings. Clinicians with various professional backgrounds provided feedback on the rating scale and its clinical usefulness for measuring several important areas of functioning, including both activity and participation. However, the extended period over many years may also be a limitation because of changes in, e.g. clinical settings, social contexts, and patient demographics. Additionally, the testing of responsiveness was performed on limited data and should be further investigated. Another limitation is that the PSF scale lacks items covering the use of the internet and social media, which are important areas today.

## Conclusion

The PSF was developed to assess functioning across different domains in persons with serious mental illness and low to moderate levels of functioning in various clinical settings. The instrument demonstrated adequate validity and reliability (in terms of internal consistency), suggesting its potential usefulness in both clinical practice and research. The subscales and items align well with the activity and participation domains of the WHO’s International Classification of Functioning, Disability and Health (ICF) framework. Nevertheless, further studies are needed to investigate additional psychometric properties and assess its usefulness in other settings.

## Supplementary Information

Supplementary Material 1.

Supplementary Material 2.

## Data Availability

The written consent from the participants does not allow for distribution of the data file to others than the research group that conducted the study. Other researchers that want access to the data may contact the first author who will answer whether the requested data may be made available in a form that does not violate the written consent from the participants.

## Abbreviations

ACT: Assertive Community Treatment
CMHC: Community Mental Health Centers
COSMIN: Consensus-based standards for the selection of health measurement instruments
GAF: Global Assessment of Functioning scale
ICF: International Classification of Functioning, Disability and Health
LSP: Life Skills Profile
PROM: Patient-reported outcome measures
PSF: Practical and Social Functioning scale
PSP: Personal and Social Performance Scale
ROM: Routine Outcome Monitoring
SD: Standard deviations
SFS: Social Functioning Scale
SOFAS: Social and Occupational Functioning Scale
SPSS: Statistical Package for the Social Sciences
WHO: World Health Organization
WHODAS: World Health Organization disability assessment schedule

## References

[1] Psychosis and schizophrenia in adults: prevention and management. NICE Guidelines. 2014. Available from: https://www.nice.org.uk/guidance/cg178/resources/psychosis-and-schizophrenia-in-adults-prevention-and-management-pdf-35109758952133. 32207892

[2] Keepers GA, Fochtmann LJ, Anzia JM, Benjamin S, Lyness JM, Mojtabai R, et al. The American psychiatric association practice guideline for the treatment of patients with schizophrenia. Am J Psychiatry. 2020;177(9):868–72. 32867516 10.1176/appi.ajp.2020.177901

[3] Long M, Stansfeld JL, Davies N, Crellin NE, Moncrieff J. A systematic review of social functioning outcome measures in schizophrenia with a focus on suitability for intervention research. Schizophr Res. 2022;241:275–91. 35217356 10.1016/j.schres.2022.02.011

[4] World Health Organization. International Classification of Functioning, Disability and Health (ICF). Geneva, Switzerland: WHO; 2001.

[5] Federici S, Bracalenti M, Meloni F, Luciano JV. World Health Organization disability assessment schedule 2.0: an international systematic review. Disabil Rehabil. 2017;39(23):2347–80. 27820966 10.1080/09638288.2016.1223177

[6] Leifker FR, Patterson TL, Heaton RK, Harvey PD. Validating measures of real-world outcome: the results of the VALERO expert survey and RAND panel. Schizophr Bull. 2011;37(2):334–43. 19525354 10.1093/schbul/sbp044 PMC3044614

[7] Burgess PM, Harris MG, Coombs T, Pirkis JE. A systematic review of clinician-rated instruments to assess adults’ levels of functioning in specialised public sector mental health services. Aust N Z J Psychiatry. 2017;51(4):338–54. 28118728 10.1177/0004867416688098

[8] Goldman HH, Skodol AE, Lave TR. Revising Axis V for DSM-IV: A review of measures of social functioning. Am J Psychiatry. 1992;149(9):1148–56. 1386964 10.1176/ajp.149.9.1148

[9] Morosini PL, Magliano L, Brambilla L, Ugolini S, Pioli R. Development, reliability and acceptability of a new version of the DSM-IV Social and Occupational Functioning Assessment Scale (SOFAS) to assess routine social functioning. Acta Psychiatr Scand. 2000;101(4):323–9. 10782554

[10] Birchwood M, Smith J, Cochrane R, Wetton S, Copestake S. The social functioning scale. The development and validation of a new scale of social adjustment for use in family intervention programmes with schizophrenic patients. Br J Psychiatry. 1990;157:853–9. 2289094 10.1192/bjp.157.6.853

[11] Alonso J, Olivares J, Ciudad A, Manresa J, Casado A, Gilaberte I. Development and validation of the Social Functioning Scale, short version, in schizophrenia for its use in the clinical practice. Actas Esp Psiquiatr. 2008;36(2):102–10. 18365790

[12] Rosen A, Hadzi-Pavlovic D, Parker G. The life skills profile: a measure assessing function and disability in schizophrenia. Schizophr Bull. 1989;15(2):325–37. 2749191 10.1093/schbul/15.2.325

[13] Rosen A, Trauer T, Hadzi-Pavlovic D, Parker G. Development of a brief form of the life skills profile: the LSP-20. Aust N Z J Psychiatry. 2001;35(5):677–83. 11551285 10.1080/0004867010060518

[14] Pedersen G, Hagtvet KA, Karterud S. Generalizability studies of the global assessment of functioning-split version. Compr Psychiatry. 2007;48(1):88–94. 17145287 10.1016/j.comppsych.2006.03.008

[15] Ruud T. Routine outcome measures in Norway: Only partly implemented. Int Rev Psychiatry. 2015;27(4):338–44. 26256034 10.3109/09540261.2015.1054268

[16] Harvey PD. Assessment of everyday functioning in schizophrenia: implications for treatments aimed at negative symptoms. Schizophr Res. 2013;150(2):353–5. 23668973 10.1016/j.schres.2013.04.022 PMC3825780

[17] Harvey PD, Sabbag S, Prestia D, Durand D, Twamley EW, Patterson TL. Functional milestones and clinician ratings of everyday functioning in people with schizophrenia: overlap between milestones and specificity of ratings. J Psychiatr Res. 2012;46(12):1546–52. 22979993 10.1016/j.jpsychires.2012.08.018 PMC3485423

[18] Ruud T, Friis S. Community-based Mental Health Services in Norway. Consortium Psychiatricum. 2021;2(1):47–54. 38601095 10.17816/CP43 PMC11003347

[19] De Vellis RF. Scale development: Theory and applications. 2nd ed. Thousand Oaks, CA: Sage Publications; 2003.

[20] Netemeyer RG, Bearden WO, Sharma S. Scaling procedures. Issues and applications. London: Sage Publications; 2003.

[21] Wallace CJ, Liberman RP, Tauber R, Wallace J. The independent living skills survey: a comprehensive measure of the community functioning of severely and persistently mentally ill individuals. Schizophr Bull. 2000;26(3):631–58. 10993403 10.1093/oxfordjournals.schbul.a033483

[22] Wallace CJ. Functional assessment in rehabilitation. Schizophr Bull. 1986;12(4):604–30. 3810066 10.1093/schbul/12.4.604

[23] Ruud T, Aarre TF, Boeskov B, le Husevag PS, Klepp R, Kristiansen SA, et al. Satisfaction with primary care and mental health care among individuals with severe mental illness in a rural area: a seven-year follow-up study of a clinical cohort. Int J Ment Health Syst. 2016;10:33. 27073414 10.1186/s13033-016-0064-8 PMC4828811

[24] Dean AG, Arner TG, Sunki GG, Friedman R, Lantinga M, Sangam S, et al. Epi Info™, a database and statistics program for public health professionals. 6 ed. Atlanta, USA: Center for Disease Control and Prevention; 1992.

[25] Bugge P, Ruud T. Evaluering av psykiatriske pasienter i kommunene. Utposten. 1995;1:22–4.

[26] Clausen H, Landheim A, Odden S, Heiervang KS, Stuen HK, Killaspy H, et al. Associations between quality of life and functioning in an assertive community treatment population. Psychiatr Serv. 2015;66(11):1249–52. 26234328 10.1176/appi.ps.201400376

[27] Mokkink LB, Prinsen C, Patrick D, Alonso J, Bouter L, Vet Cd, et al. COSMIN methodology for systematic reviews of Patient‐Reported Outcome Measures (PROMs) user manual_version 01022018. Amsterdam, The Netherlands2018.

[28] Mokkink LB, de Vet HCW, Prinsen CAC, Patrick DL, Alonso J, Bouter LM, et al. COSMIN risk of bias checklist for systematic reviews of patient-reported outcome measures. Qual Life Res. 2018;27(5):1171–9. 29260445 10.1007/s11136-017-1765-4 PMC5891552

[29] Prinsen CAC, Mokkink LB, Bouter LM, Alonso J, Patrick DL, de Vet HCW, et al. COSMIN guideline for systematic reviews of patient-reported outcome measures. Qual Life Res. 2018;27(5):1147–57. 29435801 10.1007/s11136-018-1798-3 PMC5891568

[30] Terwee CB, Prinsen CAC, Chiarotto A, Westerman MJ, Patrick DL, Alonso J, et al. COSMIN methodology for evaluating the content validity of patient-reported outcome measures: a Delphi study. Qual Life Res. 2018;27(5):1159–70. 29550964 10.1007/s11136-018-1829-0 PMC5891557

[31] Pett M, Lackey N, Sullivan J. Making Sense of Factor Analysis. Thousand Oaks, California: Sage; 2003. Available from: https://methods.sagepub.com/book/making-sense-of-factor-analysis.

[32] Cicchetti DV. Guidelines, criteria, and rules of thumb for evaluating normed and standardized assessment instruments in psychology. Psychol Assess. 1994;6:284–90.

[33] Streiner DL, Norman GR, Cairney J. Health measurement scales: A practical guide to their development and use, 5th ed. New York, NY, US: Oxford University Press; 2015. xiii, 399-xiii, p.

[34] Ruiz MA, Pardo A, Rejas J, Soto J, Villasante F, Aranguren JL. Development and validation of the “Treatment Satisfaction with Medicines Questionnaire” (SATMED-Q). Value Health. 2008;11(5):913–26. 18494753 10.1111/j.1524-4733.2008.00323.x

[35] Iversen HH, Haugum M, Bjertnaes O. Reliability and validity of the Psychiatric Inpatient Patient Experience Questionnaire - Continuous Electronic Measurement (PIPEQ-CEM). BMC Health Serv Res. 2022;22(1):897. 35821137 10.1186/s12913-022-08307-5 PMC9275271

[36] Clausen H, Ruud T, Odden S, Benth JŠ, Heiervang KS, Stuen HK, et al. Improved Rehabilitation Outcomes for Persons With and Without Problematic Substance Use After 2 Years With Assertive Community Treatment—A Prospective Study of Patients With Severe Mental Illness in 12 Norwegian ACT Teams. Front Psychiatry. 2020;11:607071. 10.3389/fpsyt.2020.607071 PMC7785822 33424668

[37] Skar-Fröding R, Clausen H, Šaltytė Benth J, Ruud T, Slade M, Heiervang KS. Associations between personal recovery and service user-rated versus clinician-rated clinical recovery, a cross-sectional study. BMC Psychiatry. 2022;22(1):42. 35042494 10.1186/s12888-022-03691-y PMC8764788

[38] Nugter MA, Engelsbel F, Bähler M, Keet R, van Veldhuizen R. Outcomes of FLEXIBLE Assertive Community Treatment (FACT) Implementation: A Prospective Real Life Study. Community Ment Health J. 2016;52(8):898–907. 25648552 10.1007/s10597-015-9831-2 PMC5108818

[39] Westen K, van Vugt M, Bähler M, Delespaul PH, Kroon H. Ontwikkeling van 2017R. Tijdschr Psychiatr. 2019. 30793270

[40] van Vugt MD, van Veldhuizen JR, Bähler M, Delespaul PH, Huffels N, Mulder CL, et al. Ontwikkeling van een modelgetrouwheidsschaal voor functie-assertive community treatment (FACT). Tijdschr Psychiatr. 2011. 21319068

[41] Baandrup L, Rasmussen J, Mainz J, Videbech P, Kristensen S. Patient-reported outcome measures in mental health clinical research: a descriptive review in comparison with clinician-rated outcome measures. Int J Qual Health Care. 2022;34(Supplement_1):ii70–97. 33404610 10.1093/intqhc/mzab001

[42] Burns T, Patrick D. Social functioning as an outcome measure in schizophrenia studies. Acta Psychiatr Scand. 2007;116(6):403–18. 17941964 10.1111/j.1600-0447.2007.01108.x

[43] Searle A, Allen L, Lowther M, Cotter J, Barnett JH. Measuring functional outcomes in schizophrenia in an increasingly digital world. Schizophr Res Cogn. 2022;29: 100248. 35444930 10.1016/j.scog.2022.100248 PMC9014442

