# Supplementary material for: The Practical and Social Functioning (PSF) scale: development and measurement properties of an instrument for assessing activity and social participation among people with serious mental illness
Source: BMC Psychiatry. 2024 Oct 16;24:693. doi: 10.1186/s12888-024-06135-x (PMC11481451; doi:10.1186/s12888-024-06135-x)
Supplement: Supplementary file 2 — Supplementary Material 2. [file 12888_2024_6135_MOESM2_ESM.pdf]

Supplementary table A. Scores and frequency distribution (%) for items of the Practical and Social Functioning scale version 3 (N=318)

	Mean	Standard Deviations	Frequency distribution (%)				
			Completely incorrect	Correct to a small degree	Partially correct	Correct to a large degree	Completely correct
B1. Wearing clean clothes and looking clean	4.30	0.94	1.9	3.5	11.6	28.6	54.4
B2. Having good personal hygiene	4.29	0.92	0.9	4.7	12.3	28.7	53.3
B3. Having well-groomed hair (and beard)	4.33	0.90	0.6	4.7	11.7	27.2	55.7
B4. Showering/bathing without help/prompting	4.53	0.80	0.6	2.5	8.2	20.2	68.5
C1. Buying/obtaining food	4.20	1.20	6.3	4.1	13.6	14.9	61.1
C2. Able to follow a recipe	4.14	1.19	5.1	6.4	14.6	17.2	56.7
C3. Making dinner	3.77	1.39	11.5	8.0	17.9	17.6	45.0
C4. Washing clothes or having them washed	4.24	1.12	3.5	7.0	11.4	18.7	59.5
D1. Managing own finances	3.86	1.33	9.1	7.2	18.9	17.6	47.2
D2. Keeping money in a safe place	4.42	0.93	2.2	3.2	8.3	22.9	63.5
D3. Paying own rent/bills/food	3.89	1.44	13.4	5.8	11.8	16.6	52.4
D4. Making money last until next payment	4.08	1.17	5.1	5.4	17.4	20.6	51.6
E1. Having friends outside health/social services	3.79	1.39	9.8	12.3	12.9	18.6	46.4
E2. Having one or more close friends	3.63	1.48	14.5	11.0	13.2	18.9	42.3
E3. Visiting other people at least monthly	3.68	1.47	13.9	9.8	15.1	16.4	44.8
E4. Being visited by other people at least monthly	3.58	1.51	16.8	9.5	14.9	17.1	41.8
F1. Talking distinctly and clearly	4.39	0.84	0.6	1.6	14.5	24.8	58.5
F2. Both staying with a topic and changing the topic	4.41	0.76	0.0	0.3	15.4	27.4	56.9
F3. Listening to others and responding to what they say	4.44	0.74	0.3	0.6	11.0	30.9	57.1
F4. Having ordinary conversations	4.33	0.85	0.3	2.9	14.6	28.3	54.0
G1. Going to movies/concerts/sports/events	2.80	1.43	24.8	21.0	22.2	13.3	18.7
G2. Having hobbies or interests	3.56	1.35	10.4	13.3	20.6	21.2	34.5
G3. Working fairly concentrated	3.55	1.16	4.5	14.3	29.6	24.8	26.8
G4. Keeping with a task for 3-4 hours	3.17	1.37	15.2	16.2	28.3	16.5	23.8
H1. Using public transport	3.52	1.59	20.4	9.2	11.1	16.2	43.0
H2. Arranging for transportation when needed	4.29	1.09	4.5	3.8	10.2	21.3	60.2
H3. Getting around when travelling on his/her own	4.03	1.24	7.0	6.3	14.3	21.3	51.1
H4. Going on vacation to other places	2.87	1.60	31.4	14.6	16.2	11.4	26.3
